# Histogram of Oriented Gradients meet deep learning: A novel multi-task deep network for 2D surgical image semantic segmentation

**DOI:** 10.1016/j.media.2023.102747

**Published:** 2023-04

**Authors:** Binod Bhattarai, Ronast Subedi, Rebati Raman Gaire, Eduard Vazquez, Danail Stoyanov

**Affiliations:** a University College London, UK; b Nepal Applied Mathematics and Informatics Institute for research (NAAMII), Nepal; c Redev Technology, UK; d University of Aberdeen, UK

**Keywords:** 41A05, 41A10, 65D05, 65D17, Semantic segmentation, Multi-task learning, Self-supervised learning, Histogram of Oriented Gradients

## Abstract

We present our novel deep multi-task learning method for medical image segmentation. Existing multi-task methods demand ground truth annotations for both the primary and auxiliary tasks. Contrary to it, we propose to generate the pseudo-labels of an auxiliary task in an unsupervised manner. To generate the pseudo-labels, we leverage Histogram of Oriented Gradients (HOGs), one of the most widely used and powerful hand-crafted features for detection. Together with the ground truth semantic segmentation masks for the primary task and pseudo-labels for the auxiliary task, we learn the parameters of the deep network to minimize the loss of both the primary task and the auxiliary task jointly. We employed our method on two powerful and widely used semantic segmentation networks: UNet and U2Net to train in a multi-task setup. To validate our hypothesis, we performed experiments on two different medical image segmentation data sets. From the extensive quantitative and qualitative results, we observe that our method consistently improves the performance compared to the counter-part method. Moreover, our method is the winner of FetReg Endovis Sub-challenge on Semantic Segmentation organised in conjunction with MICCAI 2021. Code and implementation details are available at:https://github.com/thetna/medical_image_segmentation.

## Highlights

• We investigated the Histogram of Oriented Gradients to generate pseudo-labels of images and exploited these representations as labels of an auxiliary task.
• We extended existing semantic segmentation networks to train in a multi-task framework.
• We applied our method on two challenging medical semantic segmentation data sets. Our extensive experiments demonstrate that our pipeline consistently outperforms the counter-part single task networks.

## 1 Introduction

Medical image segmentation (Lei et al., 2020b, Milletari et al., 2016, Sharma and Aggarwal, 2010, Pham et al., 2000) is an important and active research problem. The usage of semantic segmentation in several biomedical applications such as computer-assisted diagnosis (Zhao et al., 2019), robotic surgery (Colleoni et al., 2020), radiotherapy planning and follow-ups (Nemoto et al., 2020), etc., is growing day by day. Due to this reason, the research community has witnessed an unprecedented growth of research interest in this domain. There are several types of semantic segmentation problems in medical imaging. Broadly, the existing semantic segmentation tasks can be grouped into four major categories viz. organ segmentation (Hu et al., 2017), robotic-instrument segmentation (Pakhomov et al., 2019, Shvets et al., 2018), vessels segmentation (Fraz et al., 2012), and cellar and sub-cellular segmentation (Rizk et al., 2014), etc.

**Fig. 1:**
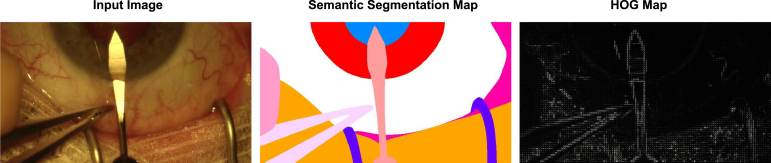
This Figure shows an input image (left) and its ground truth semantic segmentation map (left) for the primary task and the Histogram of Oriented Gradients map of the input image (right). In the HOG map, we can observe the boundary between the organs and the instruments that belong to different semantic categories. Zoom in for a better view.

After the seminal work of Krizhevsky et al. (2012) on large-scale image classification using deep convolutional neural networks, the use of deep architectures has not been limited only on computer vision (Simonyan and Zisserman, 2015, Szegedy et al., 2015, He et al., 2016); it is equally popular in medical image analysis (Suzuki, 2017, Lee et al., 2017). With the usage of deep learning algorithms, the accuracy of computer vision tasks such as classification, segmentation, and detection is improving significantly (Rawat and Wang, 2017). A similar trend has been observed on medical image analysis too (Anwar et al., 2018). We obtain the performance gain at the cost of many annotated examples (e.g. Imagenet consists of 1M annotated examples). It is evident that deep learning algorithms are data voracious and demand millions of training examples. Collecting data, in general, is time-consuming, needs experts and is also expensive. Moreover, in medical imaging, it is not only about collecting annotations as they come from highly trained experts, e.g. radiologists (e.g., MRI or CT scanner), but due to growing concerns on privacy, it is difficult to get the unlabelled examples  (Peng et al., 2021).

To improve the generalization of a model from a fixed amount of training examples, sharing the parameters between main task and auxiliary tasks (Caruana, 1997) is popular for a long time. The choice of an auxiliary task directly influences the performance of the main task. We are dealing with semantic segmentation. One of the previous studies on semantic segmentation and detection by  Dong et al. (2014) explains that semantic segmentation and detection are highly correlated tasks and often complementary in nature. MaskRCNN (He et al., 2017), one of the most popular networks in recent time, shares the parameters between detection and segmentation networks. Similarly, Takikawa et al. (2019) proposed to predict contour as an auxiliary task while training a network for semantic segmentation as the primary task. The major drawback of these methods is a need of annotated examples for both the primary and the auxiliary tasks. Collecting such a heterogeneously labelled set of training examples is even more challenging in the medical image domain.

To tackle the problem of collecting training examples with the heterogeneous set of labels, we propose to generate pseudo-labels for the auxiliary task from the hand-crafted features instead. As one can extract hand-crafted features in an unsupervised manner, generating pseudo-labels of any type of images for an auxiliary task can be done easily. To this end, we leverage the Histogram of Oriented Gradients (HOGs) (Dalal and Triggs, 2005) to generate pseudo-labels. Demarcation of the organs and surgical instruments parts belonging to a common category from unrelated ones would play a significant role in their accurate segmentation. Auxiliary tasks focusing on such aspects would help the network to learn the robust representation for semantic segmentation. Thus, we chose HOGs to generate pseudo-labels for the auxiliary task as these features are carefully designed state-of-the-art hand-crafted features for object detection  (Dalal and Triggs, 2005). However, any other type of hand-crafted features can be employed in our pipeline to extract the pseudo-labels. Fig. 1 shows the HOGs map of eye anatomy and surgical instrument. In the Figure, we can see the demarcation of a surgical instrument from eye anatomies made by the map of the Histogram of Oriented Gradients. Once, we extract the HOG features, we consider these representations as annotations of the auxiliary task and the ground truth semantic map as annotations of the primary task. We extended existing popular architectures for semantic segmentation: UNet (Ronneberger et al., 2015) and U2Net (Qin et al., 2020) to minimize the loss of both the auxiliary and primary tasks and train the network in a multi-task manner.

Use of image feature representations as a pseudo-label is growing these days. Recently, Gidaris et al. (2020) trained a deep network to predict Bag of Visual Words (BoWs) for image classification. Unlike ours, this method relied on the learned features extracted from a network trained to minimize the image rotation angle loss. In medical imaging, organs such as the eye bulb, pupils, colons, etc., are either hollow and cylindrical or rotationally invariant. Hence, the pipeline is not directly applicable in medical imaging. In addition, they trained their method to minimize the objective function of a single task, whereas we train our pipeline in a multi-task set-up. We summarize our contributions in the following points:

• We investigated the Histogram of Oriented Gradients to generate pseudo-labels of images and exploited these representations as labels of an auxiliary task.
• We extended existing semantic segmentation networks to train in a multi-task framework.
• We applied our method on two challenging medical semantic segmentation datasets: CaDIS (Grammatikopoulou et al., 2021) and Robotic Instrument Segmentation (Allan et al., 2019). Our extensive experiments demonstrate that our pipeline consistently outperforms the counter-part single task networks.

**Fig. 2:**
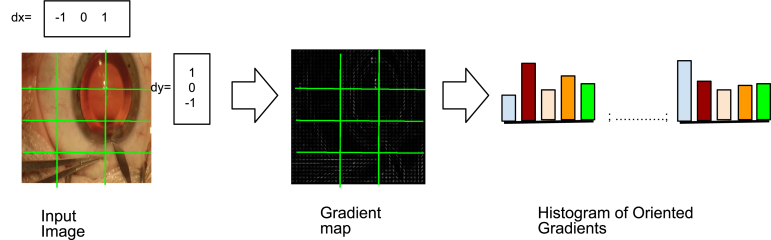
Diagram showing the pipeline to extract the Histogram of Oriented Gradients (HOGs). Zoom in for better view.

## 2 Related works

Our work falls into the category of deep multi-task learning with pseudo labels, self-supervised learning. In this Section, we summarize some of the important past works closely related to our method.

**Deep Multi-task Learning and Auxiliary-task Learning** for Semantic Segmentation: Both multi-task learning and auxiliary learning methods are explored in medical image segmentation. The subtle difference between these approaches lies in the presence or absence of a secondary task during inference time. However, these two terms are often used interchangeably in the literature. We first list some of the important multi-task works followed by auxiliary tasks.

UNet (Ronneberger et al., 2015) is one of the earliest and the most widely used deep architectures for medical image segmentation. This architecture is a supervised learning architecture and can handle only semantic maps as the ground-truth annotations. Another work on pancreas segmentation  (Roth et al., 2018) trains deep learning architecture in a multi-stage manner. It predicts the bounding box to localize the pancreas followed by fine-tuned semantic segmentation. Unlike our approach, this method uses ground truth annotations on both stages. In contrast, we rely on HOG features computed unsupervised and trained the model to minimize the losses jointly. Another work on brain lesion segmentation (Kamnitsas et al., 2017) employs 3D Convolutional Neural Network with a fully connected Conditional Random Field. Similarly,  Lei et al. (2020a) employ self co-attention to improve the performance of anatomy segmentation in whole breast ultrasound. However, these methods consider only semantic segmentation maps for ground truth. One of the recent works on tumours segmentation in 3D breast ultrasound images (Zhou et al., 2021) proposed to train CNN in multitasking fashion.  Wang et al. (2018) modified UNet architecture to jointly minimize the segmentation and classification loss in ultra-sound images.  Xie et al. (2018) trained multi-stage multitask learning framework for breast tumour segmentation in ultrasound images. Song et al. (2020) learns the parameters of network to minimize the loss for skin lesion detection, classification, and segmentation.  Chakravarty and Sivswamy (2018) trained a multi-task learning CNN for semantic segmentation and image level glaucoma classification. Another work on histopathology image analysis (Qu et al., 2019) trained a multi-task network for nucleus classification and segmentation. All of these methods need ground truth annotations for both the main task (semantic segmentation) and auxiliary tasks. Whereas, in our case, we have annotations for the primary task and generate pseudo-labels for the auxiliary task.

We present here some of works on auxiliary task. Zhang et al. (2014) proposed to estimate head pose as an auxiliary task for improving the facial landmark identification. Similarly, in detecting indoor objects, Mordan et al. (2018) leveraged the effectiveness of scene labels prediction and depth and surface orientation evaluation at pixel level. For semantic segmentation of medical images, Feyjie et al. (2020) added an auxiliary task of image denoising. In a recent work to diagnose COVID-19 from other pneumonia and normal control, Li et al. (2021) trained the model with an auxiliary task of contrastive learning to learn transformation invariant representations. The addition of subsidiary task has been proven effective in boosting the performance of the network for the main task.

**Self-supervised Learning:** In Self-supervised learning, the annotations for the pre-text tasks are generated in an unsupervised manner. In general, the parameters of a CNN are learned to minimize the loss of pre-text tasks followed by fine-tuning of the parameters for the downstream tasks. Several different ways are investigated in the past years to generate the annotations of pre-text tasks. These includes, image rotation angle (Gidaris et al., 2018), colourization (Zhang et al., 2016), image-patch context (Pathak et al., 2016), in-painting (Pathak et al., 2016), etc. These methods mostly pivot on the geometric transformations of the images. What kind of pre-text task is going to be the most useful for the end-task is still an open research problem. Recently, Gidaris et al. (2020) proposed to learn the representations by predicting the visual Bag of Words (BoW). This method, closest to ours, rely on visual features to generate the pseudo-labels. As we mentioned before, they compute BoWs from the visual representations extracted from model trained to minimize the rotation angle of an image. Thus, this approach is not directly applicable to our applications as most of the organs such as eyes, eye-bulb exhibit rotationally invariant shape. Unlike most of the self-supervised pipeline, we propose to minimize the loss of end-task and pre-text task jointly.

**Fig. 3:**
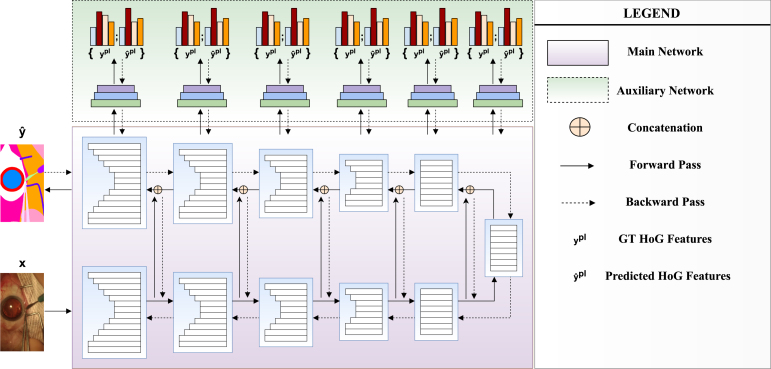
This diagram shows the overall proposed framework. In the Figure, the main network corresponds to semantic segmentation network (e.g. U2Net), while the auxiliary network is our contribution to extend the single task network to a multi-task network. Training examples in triplet, i.e. input image, ground truth semantic map and pseudo-label computed from HOGs, are fed into the network and train the network jointly.

## 3 Proposed method

In this Section, we present our pipeline in detail. We start with the description of HOGs followed by the generation of pseudo-labels for the auxiliary task. Afterwards, we explain our approach to extend a single-task semantic segmentation network to a multi-task network. Finally, we explain the overall objectives.

We have a scenario X×Y where X represents input image space and Y represents output semantic map space. Our goal is to learn a function f:X→Y with a given training examples $T=\{\left(x_{1},y_{1}\right),\left(x_{2},y_{2}\right)\ldots \left(x_{i},y_{i}\right)\ldots \left(x_{N},y_{N}\right)\}\subset X\times Y$. In the training set T, N is total number of training examples, $x_{i}\in R^{\left(W\times H\times C\right)}$, $y_{i}\in R^{\left(W\times H\right)}$, where, W,H,C represents width, height, and total number of channels in an image respectively. Our contribution lies in generating extra annotations of the images in an unsupervised way and extending the single task semantic segmentation network to train in a multi-task manner to improve the performance of semantic segmentation. We make use of HOGs to extract the pseudo-annotations of an image.

### 3.1 Histogram of oriented gradients as pseudo labels

It is proven that the HOGs (Dalal and Triggs, 2005) were one of the most powerful hand-features on computer vision and medical image analysis especially for detection before the advent of data driven feature extraction methods such Alexnet (Krizhevsky et al., 2012), ResNet (He et al., 2016), and UNet (Ronneberger et al., 2015). In this paper, we use HOGs for a novel cause i.e. to extract the pseudo-labels of the images. To compute HOGs from an image, first of all, we crop and resize the images to the desired dimensions of width, W and height, H. We further divide the images into a non-overlapping image patches of width w, and height h, resulting the total number of patches of ⌊W/w⌋×⌊H/h⌋. For each of the patches, we run 1-D discrete derivative masks centred around a pixel in both the horizontal and vertical directions. $d_{x}=\left[1,0,-1\right]$ and $d_{y}={\left[1,0,-1\right]}^{T}$ are horizontal and vertical filtering kernels respectively. We run these filters on all the pixels of every image patches as shown in Fig. 2.

After applying the kernels centred on every pixels, we compute the histogram of gradients for all the patches and append them together. Gradients are computed as $\arctan\left(\frac{d_{y}}{d_{x}}\right)$, and the gradients are assigned to the nearest bin. The histogram can have k number of bins with angle ranging from 0 to 180 degrees. The magnitude of the gradient is computed as $\sqrt{d_{x}^{2}+d_{y}^{2}}$. This magnitude of the gradients encodes the frequency of a bin of the gradient taken into consideration. In this manner, we estimate the histogram of oriented gradients in every patch. The number of the bins and the patches determine the dimension of the HOGs and are the hyper-parameters in our study. We present their studies in Experimental Section in depth. We concatenate the HOGs for all the patches of an image, and the final representations of HOGs are the pseudo-label, $y^{pl}$ of the image. We augment the pseudo-label on the given training set. Thus, the training set with augmented pseudo-labels become ${\left\{\left(x_{i},y_{i},y_{i}^{pl}\right)\right\}}_{i=1}^{i=N}$ which we use to train the semantic segmentation network in multi-task setup.

### 3.2 Multi-task semantic segmentation with pseudo labels

For an input image x with the ground truth semantic segmentation map y and its pseudo-label $y^{pl}$, we train a semantic segmentation network in a multi-task learning fashion. The primary task for us is to predict the semantic map and the secondary task is to regress the Histogram of Oriented Gradient (HOG) features. To predict the semantic map we employ categorical cross-entropy loss and minimize mean squared loss to predict the HOG features. As mentioned before, UNet and U2Net are two most popular and the powerful semantic segmentation networks in medical imaging. However, these networks are originally designed to support semantic map as only ground truth. Thus, these networks cannot readily handle our heterogeneously labelled training examples. To enable them to handle pseudo-labels and share the parameters between these tasks, we proposed to add a regression unit with two convolutional layers and a fully connected layer on every layers of the decoder side on U2Net as shown in Fig. 3. On UNet, we added only one such unit on bottleneck. It is because, UNet has relatively less parameters compared to U2Net. In Fig. 3, the lower block depicts the U2Net architecture and the upper block shows the regression units we introduced in the architecture. The regression units learn the parameters predicts HOGs correctly. In the similar manner, we plugged in regression units on UNet. Compared to UNet, U2Net is also an hourglass architecture where each layer consists of a UNet. We learn the parameters of the whole architecture to minimize the following objective (see Table 1).

(1) $$L=\frac{1}{N}\overset{i=N}{\underset{i=1}{\sum }}\alpha L_{ce}\left(x_{i},y_{i}\right)+\beta L_{HOG}\left(x_{i},y_{i}^{pl}\right)$$

In Eq. (1), $L_{ce}$ is the primary task loss i.e. minimization of cross-entropy loss to predict the ground truth mask correctly. Whereas, $L_{HOG}$ is loss of secondary task to predict the HOGs of the input image. We minimize the mean squared error between the predicted and ground truth HOG features. α and β are two hyper-parameters to weight the contributions of each of the losses to best generalize the model parameters on unseen data for semantic segmentation. We fine-tune these parameters by doing cross-validation on validation set. The details are on Section 4.

**Table 1:** Architecture of the auxiliary task network to regress HOGs.

Input shape	Operations
(3,h,h)	Conv(3,3,1), ReLU(), MaxPool2d(2,2)
(3, $\frac{h}{2},\frac{h}{2}$)	Conv(3,3,1), ReLU(), MaxPool2d(2,2)
(3, $\frac{h}{4},\frac{h}{4}$)	Flatten()
($3\times \frac{h}{4}\times \frac{h}{4}$)	Linear(504)

## 4 Experiments

### 4.1 Datasets

We evaluated our methods on two different publicly available challenging data sets with diverse characteristics. CaDIS data set (Grammatikopoulou et al., 2021) was released in MICCAI 2020 in one of the EndoVis challenges. It consists of 25 surgical videos. Each video frame is annotated broadly into eye anatomies, surgical instruments, and miscellaneous categories. Based on the granularity of the segments,  Grammatikopoulou et al. (2021) designed the challenge into three different tasks. Task 1 consists of 8 different segments: four eye anatomies, three misc objects, and one instrument category. In Task 2, the instrument category is further split into nine classes, resulting in 17 different categories. Finally, in Task 3, there is an increase in granularity on the handles of the surgical instrument. This further increase in granularity resulted in 25 different categories to segment. There are 3550 annotated frames in train set, 534 in validation set, and 586 are in test set.

Another data set on which we evaluated our method is Robotic Instrument Segmentation (Allan et al., 2019). This data set is publicly available for research since MICCAI 2017 challenge. The main task on this data set is to segment surgical instruments from the background. Based on the granularity of segmentation of the parts of the surgical instruments, three tasks were designed in the challenge. Task 1 is to segment the instruments as a whole from the rest of the background. Similarly, the challenge in Task 2 is to segment the instruments parts into wrist, jaw, and shaft and distinguish the instrument from the background. Finally, Task 3 further segments the instrument into seven types and segregates it from the background. There are 10 different folds of videos in total. Following the evaluation protocol described on Allan et al. (2019), we report performance on folds 9 and 10 and train on rest of the videos.

### 4.2 Baselines architectures

We took UNet (Ronneberger et al., 2015) and U2Net (Qin et al., 2020), two representative architectures, for semantic segmentation and employed our method on these two architectures. Since our method is generic in nature, we can easily extend to other architectures. UNet is one of the most widely used architectures in medical image segmentation. It is a lightweight architectures consisting of encoder and decoder. Encoder consists of convolutional and pooling layers that map high-dimensional images into low-dimensional latent space. Decoder feeds in the latent representations of the image and learns the parameters to predict the correct semantic maps. There are skip connections from encoder layers to decoder layers.

U2Net is another recently proposed architecture with state-of-the-art performance on multiple computer vision semantic segmentation benchmarks. Similar to UNet, this is an hourglass architecture with skip connections between the encoder and decoder layers. Compared to UNet, U2Net consists of UNet like structures in every layer of encoders and decoders and also known as UNet inside UNet. Thus, the learning parameters in this architecture are much higher than UNet.

### 4.3 Evaluation metrics

We used mean Intersection of Union (mIoU) to compare the quantitative performance. Intersection of Union (IoU) is computed as follows:

$$\text{IoU}=\frac{\text{true positive}}{\text{true positive + false positive + false negative}}$$

In addition to this, we also present extensive qualitative analysis to make the comparisons.

**Table 2:** Summary of quantitative performance comparison on CaDIS data set.

Task	# Classes	Validation set mIoU			Test set mIoU		
		MICCAI’21	U2Net	+HOG (Ours)	MICCAI’21	U2Net	+HOG (Ours)
1	8	**86.7**	84.9	85.5	**83.7**	80.2	81.4
2	18	72.7	83.8	**84.1**	70.6	77.8	**80.2**
3	26	66.6	82.1	**83.0**	59.2	78.2	**78.4**

**Table 3:** Summary of quantitative performance comparison on Robotic Instrument Segmentation data set.

Task	# Classes	mIoU on test video 9					mIoU on test video 10				
		MICCAI’17	U2Net	+Contour	+HOG${}_{bn}$	+HOG	MICCAI’17	U2Net	+Contour	+HOG${}_{bn}$	+HOG
1	2	87.7	94.2	95.0	95.0	**95.6**	91.7	96.0	96.15	95.7	**96.2**

2	4	73.6	70.8	74.3	74.1	**75.8**	80.7	84.1	83.9	83.9	**84.4**

3	8	35.7	57.9	**66.2**	56.3	65.4	79.1	89.4	**92.9**	90.6	91.3

**Fig. 4:**
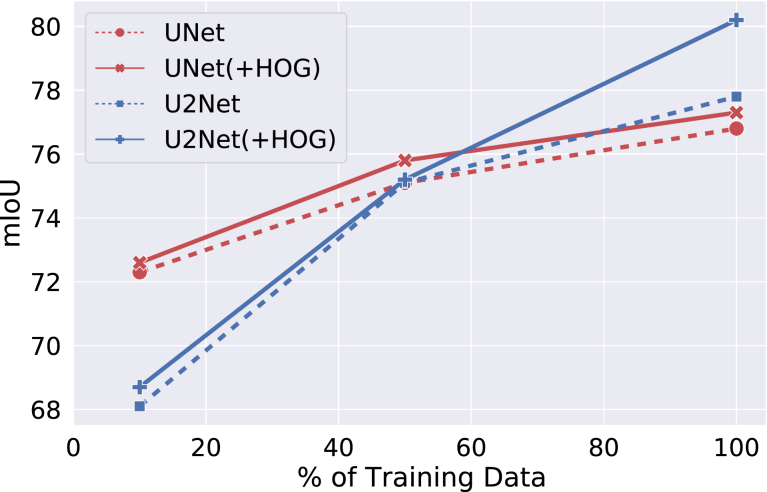
Performance comparison with varying sizes of training data on test dataset of CaDIS Task 2 segmentation.

### 4.4 Implementation details

To compute HOGs from the images, inspired from the original paper on HOG (Dalal and Triggs, 2005), we resized the image to the dimension of (64 × 128). Other parameters that determine the size of HOG features are the number of histogram bins and patch size. We set the number of bins of the histogram (k) to 6 to set each bin with an angle range of 30. Initially, we took a patch of size 16 × 16, which outputs a vector of dimension 504. Similarly, setting the patch size to 12 × 12 and 8 × 8 gives us HOG features of dimensions 864 and 2520, respectively.

We implemented our algorithms on PyTorch framework. For optimization, we employ Adam Optimizer. We set the initial learning rate to 2e–4 and scaled it by a factor of 0.5 in every 50k iteration. We train our algorithms for 150k iterations and validate every 1k iterations.

**Fig. 5:**
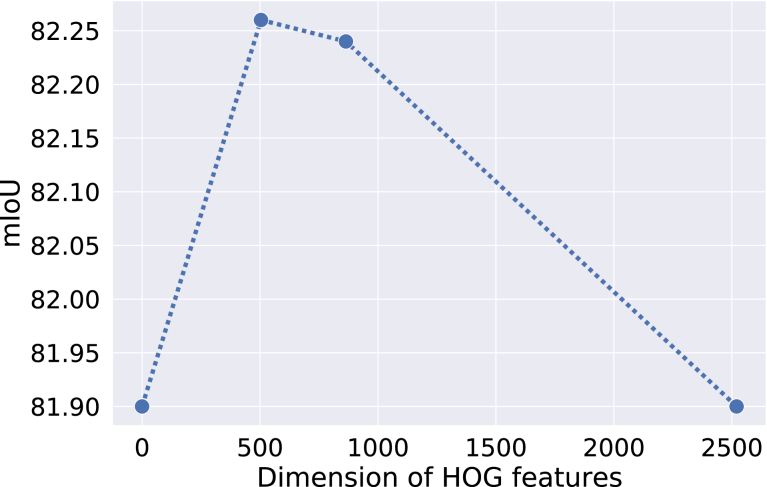
Performance comparison with varying dimensions of HOG features.

**Fig. 6:**
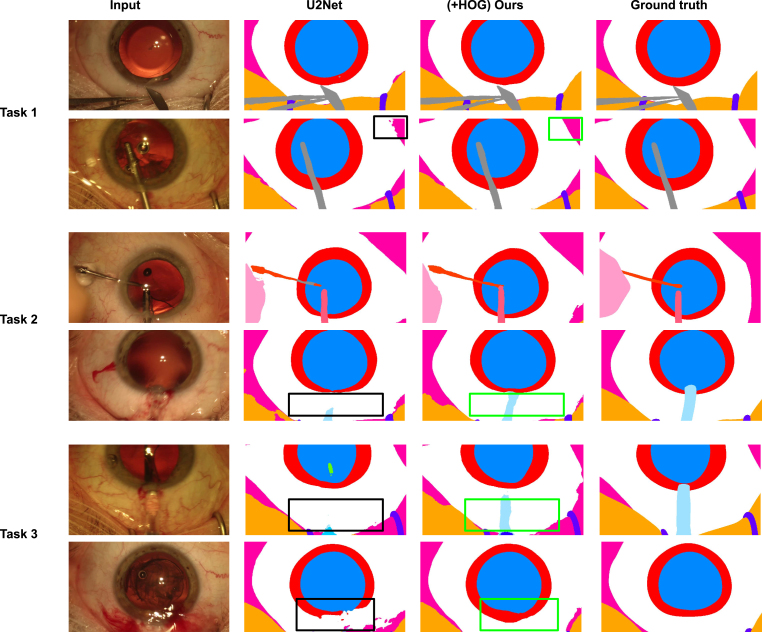
Qualitative comparison between the proposed method with its counter-part architecture U2Net on three different tasks. First two rows represent examples from Task 1, the middle two rows, and the last two rows are examples from Task 2 and Task 3 respectively.

**Fig. 7:**
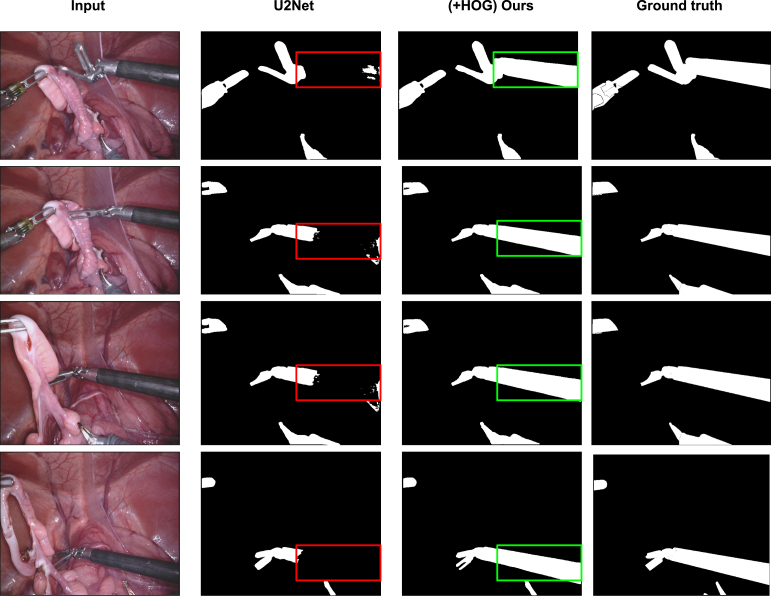
Qualitative comparison between before and after applying our method on U2Net in the Task 1 of robotic instrument segmentation challenge held in MICCAI 2017.

**Fig. 8:**
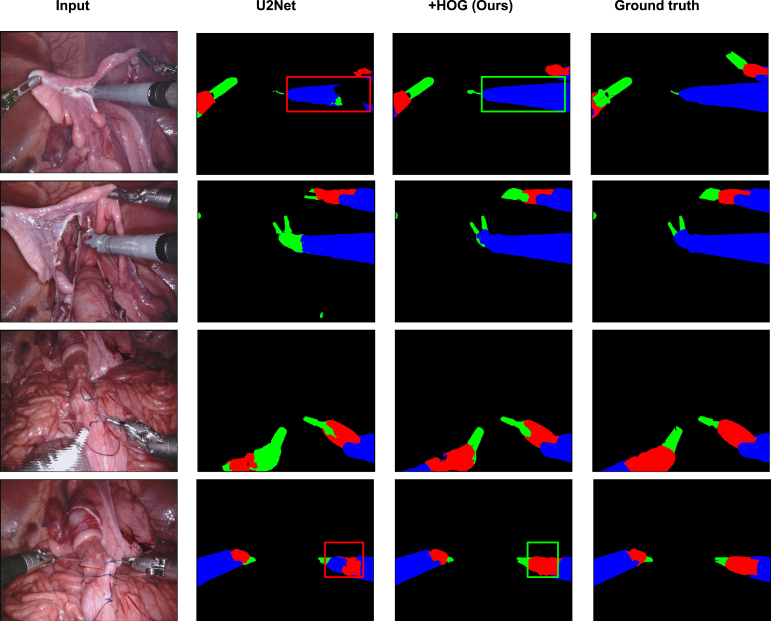
Qualitative comparison between before and after applying our method on U2Net in the Task 2 of robotic instrument segmentation challenge held in MICCAI 2017.

**Table 4:** Ablation study on weights of losses. The number inside the bracket represents the dimension of HOG features. The mIoU reported for UNet network is on CaDIS segmentation task 2 validation dataset and that for U2Net network is on Robotic Instrument segmentation task 1 test video sequence 9.

Weight of losses		mIoU	
α	β	UNet(504)	U2Net(504)
0.01	1.0	81.2	94.7
0.1	1.0	82.1	95.4
1.0	1.0	**82.3**	**95.6**
1.0	0.1	81.7	94.8
1.0	0.01	81.4	94.5

### 4.5 Hyper-parameters selection

There are two critical sets of hyper-parameters in our proposed pipeline. The first one is the weights of the primary loss (α) and the secondary loss (β) as shown in Eq. (1). Another hyper-parameter is the dimension of HOG features. We estimated the values of these hyper-parameters by doing cross-validation on Validation Set. As the detection and segmentation tasks are highly co-related (Dong et al., 2014), we set the α and β to 1.0 to give them equal importance. Fig. 11 summarizes the minimization of both losses. From the Figure, we can infer that the correct prediction of HOG features is equally important to that of semantic segmentation for the overall performance. Then, we fine-tune the dimension of HOG features. We observe the highest performance when the dimension HOG features is 504. Afterwards, we fix the dimension to 504 and vary the weights of losses. Table 4 summarizes the cross-validation for weighing the contributions of the proposed losses on CaDIS and Robotic Instrument datasets. We observed that setting equal contribution to the losses gives the optimal performance. We observed a similar trend on another benchmark too. This outcome also highlights the significance of the proposed auxiliary loss in our pipeline. We set the values of α and β equal to 1 in the rest of the experiments. Similarly, Fig. 5 shows the performance on CaDIS Validation Set with varying the dimension of the HOGs. We can see the highest performance with the dimension of 504, which we set for the rest of the experiments.

**Table 5:** Category-wise mIoU on Robotic Instrument Segmentation dataset for parts based segmentation.

Instrument parts	Methods			
	U2Net	+contour	+HOG${}_{bn}$	+HOG (Ours)
Shaft	85.7	**90.4**	89.8	90.0
Wrist	68.9	71.5	71.6	**72.2**
Jaw	60.9	63.9	64.8	**65.5**

**Table 6:** Comparison of categorical performance for Task 2 on CaDIS dataset.

Classes	Methods	
	U2Net	+HOG (Ours)
Pupil	94.2	**94.3**
Surgical Tape	82.6	**87.0**
Hand	84.6	**86.0**
Eye Retractors	84.6	**85.3**
Iris	**85.1**	84.3
Skin	64.7	**69.5**
Cornea	**92.9**	92.8
Cannula	43.5	**45.5**
Cap. Cystotome	36	**47.6**
Tissue Forceps	62.9	**69.9**
Primary Knife	80.1	**81.8**
Ph. Handpiece	77.7	**79.2**
Lens Injector	73	**73.5**
I/A Handpiece	70.4	**71.0**
Secondary Knife	52.3	**63.0**
Micromanipulator	**57.7**	52.8
Cap. Forceps	**16.3**	14.4

### 4.6 Quantitative evaluations

Here, we present the outcomes from our extensive experiments on two different data sets: CaDIS and Robotic Instrument Segmentation. As mentioned before, each of the benchmarks consists of three tasks resulting in six different tasks from two data sets. We extended our method on two popular baseline architectures: UNet and U2Net. We evaluate the empirical performance on the mean Intersection of Union (mIoU).

**Comparison on varying training data size:** Compared to U2Net, UNet is more efficient but is less accurate. We evaluate both the architectures on CaDIS Task 2. We choose this task due to the good trade-off of granularity and the number of training examples per category. In this experiment, UNet and U2Net obtained 81.9% and 83.75% mIoU, respectively on full training data. We also took a different proportion of training examples and compared the performance of both UNet and U2Net with/out the auxiliary task to predict HOGs. Fig. 4 summerizes our experiments. Our techniques to extend both the networks to multi-task networks improve the performance consistently. This gain in performance also shows that our method equally generalizes on varying sizes of training examples. For experiments on the remaining tasks from both the data sets, we decided to choose U2Net as our baseline architecture as its performance is clearly superior to UNet.

**Fig. 9:**
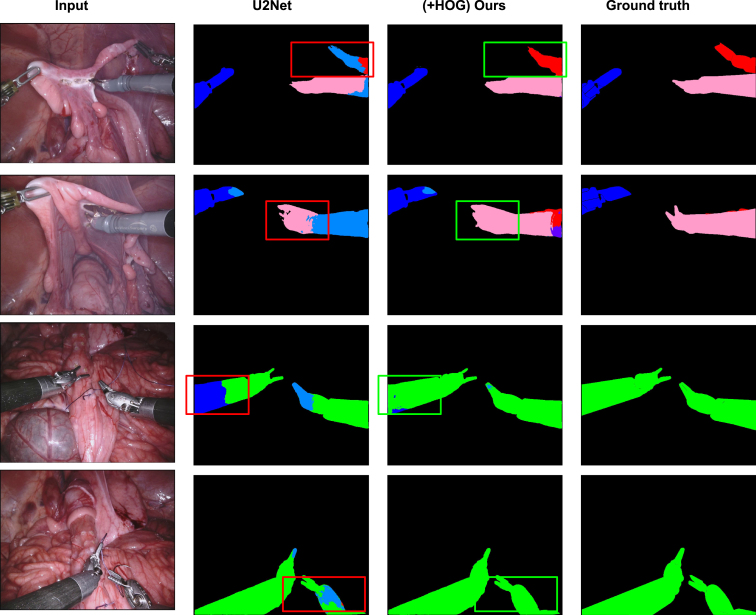
Qualitative comparison between before and after applying our method on U2Net in the Task 3 of robotic instrument segmentation challenge held in MICCAI 2017.

**Comparison on CaDIS dataset:**Table 2 summarizes the performances of three different tasks on CaDIS data set. We have compared our performance with the winner of the MICCAI 2021 challenge and U2Net. From the Table, we can see that our method consistently outperforms the U2Net on both the validation set and the set. The class-wise mIoU reported in Table 6 further validates the significance of the proposed pipeline over its counterpart. Similarly, out of 6 different scenarios, our method obtained the highest mIoU on 4 cases, slightly lagging behind the winner of MICCAI’21 challenge on Task 1. Compared to Task1, on Task 2 and Task 3, the mIoU of the winning method on MICCAI’21 dropped by a large margin (−20%). In contrast, our cases have a slight drop in performance (−2.0%). This shows the robustness of the proposed pipeline over the increase in the granularity of the segmentation tasks.

**Comparison on Robotic Instrument Segmentation dataset:**Table 3 details the performance comparison on Robotic Instrument Segmentation. We followed the evaluation protocol presented on the challenge paper and compared our performance with the winner model. In every task, our method obtained the highest mIoU surpassing the winning team’s performance and our baseline U2Net by a large margin. With the increase in the granularity in the segmentation task, the mIoU of the winner method drops by up to −50%. At the same time, the drop in our method is only up to −30.2%. Again, this is yet another evidence for our method being robust compared to the contemporary methods. Similarly, our method and the baseline predicting contour as an auxiliary task outperform in 4/6 and 2/6 cases, respectively. The empirical performance between these methods looks comparable. Compared to predicting contour, the advantage of our approach is that we can generate pseudo labels in an unsupervised manner, but the contour-based method demands ground truth semantic segmentation. Further looking into the class-wise performance in Task 2, our method outperforms the competitive baseline in 2/3 cases (see Table 5).

**Comparisons with the State-of-the-art Methods:**

**Fig. 10:**
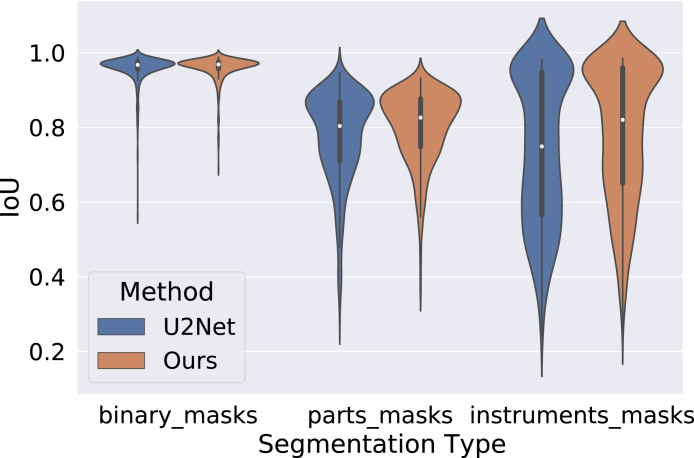
Violin plot showing the distribution of IoU of test images (sequences 9 and 10) on all three tasks of the Robotic Instrument Segmentation dataset. A small white dot on each violin represents the median IoU.

**Fig. 11:**
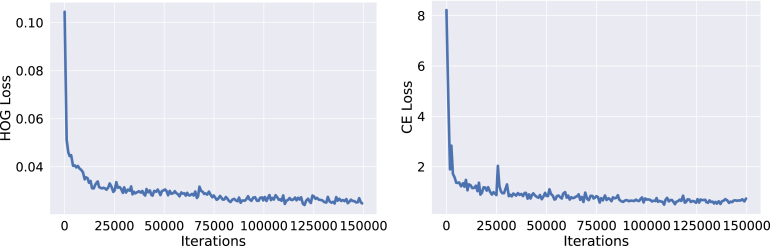
Training curve of our method on CaDIS task 3.

In Table 7, we report the mIoU of different methods on the Robotic Instrument Segmentation dataset for instrument type segmentation. UNet  (Ronneberger et al., 2015), the popular benchmark network for medical image segmentation, achieved 46.1%. The winner of the 2017 Robotic Instrument Segmentation Challenge, TernausNet (Iglovikov and Shvets, 2018), which has similar architecture to UNet but employs a VGG16 network as an encoder, obtained a mean score of 56.4%. The mIoU score of DeepLabV3+  (Chen et al., 2018) is 64.2%. This network implements atrous convolution, which provides the benefit of long-range contextual information. Likewise, mIoU scores of LinkNet (Chaurasia and Culurciello, 2017), PAN (Li et al., 2018), PAANet (Ni et al., 2020), and DANet (Fu et al., 2019) are 56.0%, 64.1%, 64.2%, and 63.1% respectively. SurgiNet (Ni et al., 2022) is the state-of-the-art method on Robotic Instrument Segmentation to date. is SurgiNet (Ni et al., 2022). This method proposed to train neural network architecture with a double attention module. The mIoU obtained by this method is 66.3%. Our method obtains 70.2% attaining the new state-of-the-art (see Table 8).

**Table 7:** Performance comparison of our proposed method with SurgiNet and various methods of EndoVis 2017 Robotic Instrument Segmentation Challenge on instrument type segmentation. We report the results from Ni et al. (2022).

Methods	mIoU
UNet (Ronneberger et al., 2015)	46.1
TernausNet (Iglovikov and Shvets, 2018)	56.4
LinkNet (Chaurasia and Culurciello, 2017)	56.0
PAN (Li et al., 2018)	64.1
PAANet (Ni et al., 2020)	64.2
DANet (Fu et al., 2019)	63.1
DeepLabV3+ (Chen et al., 2018)	64.2
SurgiNet (Ni et al., 2022)	66.3
U2Net+HoG (**Ours**)	**70.2**

**Table 8:** Performance comparison of our proposed method TernausNet on 10 test sequences of instrument type segmentation.

	TernausNet	Ours
Dataset 1	**53.8**	42.3
Dataset 2	74.3	**75.4**
Dataset 3	67.6	**83.9**
Dataset 4	**89.2**	61.7
Dataset 5	43.3	**54.8**
Dataset 6	60.6	**61.8**
Dataset 7	49.4	**64.3**
Dataset 8	31.4	**52.6**
Dataset 9	46.2	**65.4**
Dataset 10	52.9	**91.3**

### 4.7 Qualitative evaluations

We did not limit our experiments to quantitative evaluations only. To deeper understand our method’s role in improving the performance of existing architecture such as U2Net, we performed an extensive qualitative analysis. Fig. 6 shows the qualitative comparisons of Task 1, Task 2, and Task 3 on CaDIS data set. The bounding boxes locate some of the representative regions on the eye and the surgical instrument where U2Net fails, but our method correctly segments it. From these locations, we can see that the characteristics of HOGs to identify the organs and tools boundary play a crucial role in correctly segmenting the organs and the semantic parts of the surgical tools.

Similarly, Fig. 7, Fig. 8, Fig. 9 show the qualitative comparison of Task 1, Task 2, and Task 3 on robotic instrument segmentation. In these qualitative analyses, we observe the similar trends that were seen on CaDIS data set. As we can see from these analysis, U2Net struggles quite a lot on boundary regions. Our method enables correct segmentation on such regions that we can see in our qualitative comparisons. The red bounding boxes on the Figures locates the failed cases by the baseline, whereas the green bounding boxes show the correction made by our method.

In order to observe the distribution of IoU of individual test images, we show violin plots in Fig. 10 on all the tasks for Robotic Instrument Segmentation. From left to right, the figure shows the violin plots of Tasks 1, 2, and 3, respectively. These plots also demonstrate the robustness of our method over the counter-part baseline. The median mIoU(represented by a white dots in 10) of our method is higher than that of the counter-part in each task.

## 5 Conclusions and future works

In conclusion, we present a novel multi-task deep learning framework for medical image segmentation. We generate the annotations of the auxiliary task in an unsupervised manner by leveraging the Histogram of Oriented Gradients features of images as their labels. We train the deep network jointly to minimize the losses of both the primary task, semantic segmentation, and the auxiliary task. Our extensive qualitative and quantitative results on two challenging medical image segmentation benchmark datasets, CaDIS and EndoVis 2017 Robotic Instrument Segmentation, show that the proposed pipeline’s performance is superior to its counterpart single task network. The inclusion of HOG feature prediction as an auxiliary task enforces the network to learn more meaningful representations to distinguish boundaries among different classes in the shared layer. Experiments with different baseline architecture like UNet and U2Net validate the generalizability of our approach. Moreover, our proposed method achieved the best performance in most segmentation tasks of two benchmark datasets.

As we can obtain HOG features in an unsupervised way, its applicability in medical image analysis, where annotating images is both costly and time-consuming, can be further extended, which we aim to investigate in the future. Furthermore, we plan to explore the higher-order statistics of hand-crafted features such as Fisher Vectors as annotations of images to train the multi-task deep semantic network.

## Declaration of Competing Interest

The authors declare that they have no known competing financial interests or personal relationships that could have appeared to influence the work reported in this paper.

## Data Availability

We made code public. The link is shared in the paper.
